# Direct observation of symmetrization of hydrogen bond in δ-AlOOH under mantle conditions using neutron diffraction

**DOI:** 10.1038/s41598-018-33598-2

**Published:** 2018-10-19

**Authors:** Asami Sano-Furukawa, Takanori Hattori, Kazuki Komatsu, Hiroyuki Kagi, Takaya Nagai, Jamie J. Molaison, António M. dos Santos, Christopher A. Tulk

**Affiliations:** 1 J-PARC Center, Japan Atomic Energy Agency, Tokai-mura, Ibaraki, 319-1195 Japan; 2 Geochemical Research Center, Graduate School of Science, The University of Tokyo, Tokyo, 113-0033 Japan; 3 Department of Earth and Planetary Sciences, Faculty of Science, Hokkaido University, Sapporo, 060-0810 Japan; 4 Neutron Scattering Division, Oak Ridge National Laboratory, Oak Ridge, Tennessee 37831 USA

## Abstract

At ambient pressure, the hydrogen bond in materials such as ice, hydrates, and hydrous minerals that compose the Earth and icy planets generally takes an asymmetric O-H···O configuration. Pressure significantly affects this configuration, and it is predicted to become symmetric, such that the hydrogen is centered between the two oxygen atoms at high pressure. Changes of physical properties of minerals relevant to this symmetrization have been found; however, the atomic configuration around this symmetrization has remained elusive so far. Here we observed the pressure response of the hydrogen bonds in the aluminous hydrous minerals δ-AlOOH and δ-AlOOD by means of a neutron diffraction experiment. We find that the transition from *P*2_1_*nm* to *Pnnm* at 9.0 GPa, accompanied by a change in the axial ratios of δ-AlOOH, corresponds to the disorder of hydrogen bond between two equivalent sites across the center of the O···O line. Symmetrization of the hydrogen bond is observed at 18.1 GPa, which is considerably higher than the disorder pressure. Moreover, there is a significant isotope effect on hydrogen bond geometry and transition pressure. This study indicates that disorder of the hydrogen bond as a precursor of symmetrization may also play an important role in determining the physical properties of minerals such as bulk modulus and seismic wave velocities in the Earth’s mantle.

## Introduction

The existence of hydrogen in the Earth’s mantle is evidenced by the presence of high-pressure ice^1^, hydrous minerals^2^, and nominally anhydrous minerals with high water content^3^ as inclusions in superdeep diamonds. Hydrogen, which has only one electron, is anchored to minerals by a hydrogen bond (H-bond). Because of the distinct bond property of H-bond as compared to that of the covalent bond that forms rigid frameworks of the minerals, the incorporation of hydrogen significantly influences the physical properties of mineral^4^.

At ambient pressure, the hydrogen bond generally takes an asymmetric O-H···O configuration that comprises a short O-H covalent bond and a long H···O hydrogen bond. The O-H bond length is around 1.0 Å whereas the H···O bond length is around 1.8 Å in a moderate strength of H-bond. By calculating the proton potential with respect to the distance between the two oxygen atoms, Holzapfel (1972)^5^ indicated that the hydrogen in high-pressure ice will be centered along the O···O line at a point where the double-well proton potential merges into a single minimum under compression. This process is the so-called symmetrization of the H-bond. (In this paper we use the term “symmetrization” to indicate the model in which proton locates at the center position between the two oxygen atoms in a statistical view. The model with fully disordered hydrogen bond between two off-centered sites with half occupancy in each is refereed as a “disordered” phase in this paper following the previous studies on ice although it is crystallography symmetric as well.) Considerable effort has been expended to investigate the pressure-induced symmetrization of the H-bond; regardless, most of the studies that have been conducted so far are based on indirect methods such as spectroscopy^6–9^ and X-ray diffractions.

**Figure 1:**
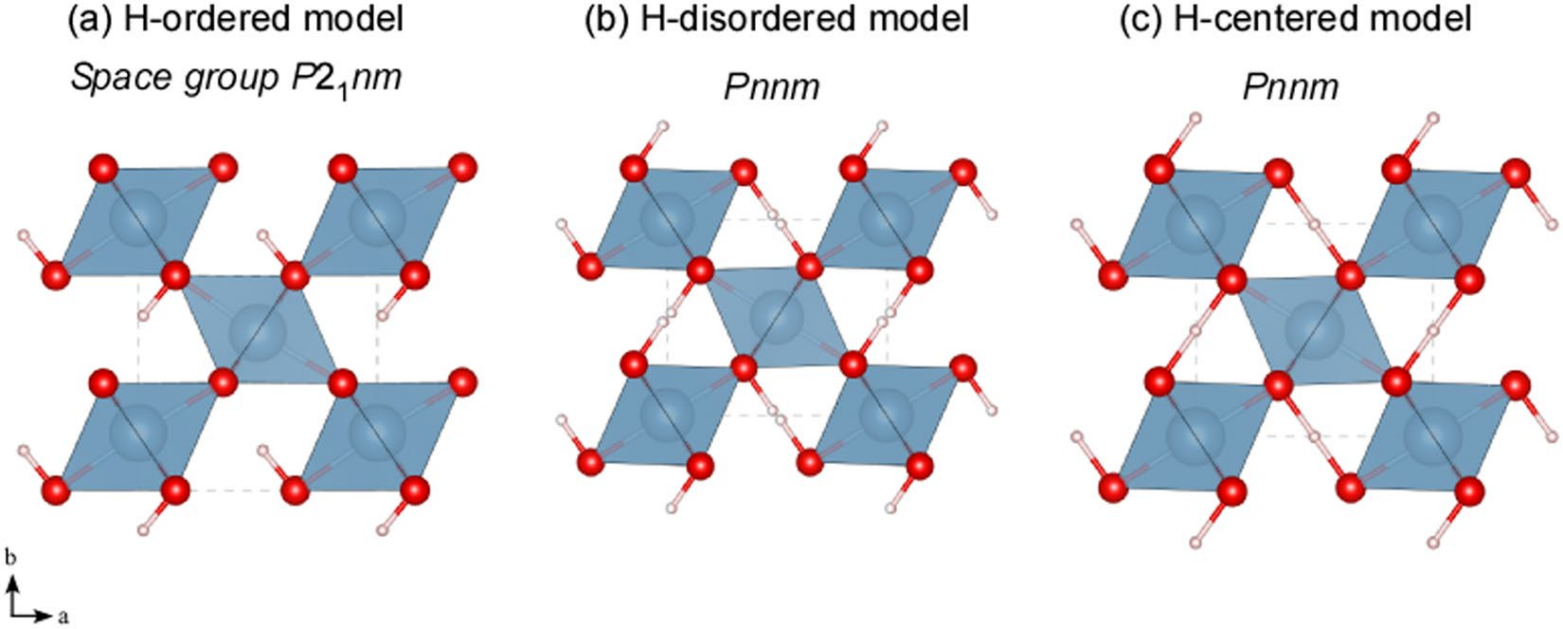
Structure models of δ-AlOOH (**a**) at ambient pressure with hydrogen-ordered model, (**b**) hydrogen disordered, and (**c**) centered model. AlO_6_ octahedra (blue) share edges and form a single chain along the *c*-axis. Each chain connects with neighbors via corners, and these chains build a distorted-rutile-type framework. At ambient pressure (**a**), hydrogen (pink spheres) is located in the tunnel between the octahedral chains at asymmetric positions between two oxygen atoms (red sphere)^25,43^. In this case, the Al atom sits at an off-center position in the octahedron, reflecting the different electrostatic charge between the donor and acceptor of the H-bond. By contrast, the Al atom sits at the center of the octahedron in the H-disordered (**b**) and the centered models (**c**).

Further, the H-bond symmetrization is predicted to occur in minerals at the mantle conditions. The first theoretical prediction of the H-bond symmetrization in minerals was conducted on the aluminous hydrous mineral, δ-AlOOH^10–13^ (Fig. 1), which reported that the symmetrization occurs at around 30 GPa. This hydrous mineral is stable over an extremely wide pressure range beyond 134 GPa and 2000 K; thus, it is considered to be one of the important hydrogen carriers to the Earth’s core-mantle boundary^14,15^. It was also indicated that the H-bond in other hydrous minerals, such as dense hydrous magnesium silicates phase D^16^, phase H^17^, and FeOOH^18^, that are stable in the lower mantle conditions, will undergo symmetrization. Notably, the change in bonding nature owing to symmetrization induces a change in the physical properties of the mineral. Theoretical studies have indicated that symmetrization triggers an increase in the bulk modulus and that it modifies the seismic wave velocity in δ-AlOOH^10–12^. Subsequent experimental studies observed a stiffening behavior at around approximately 10 GPa^19^ and an anomalous increase in sound velocities over an extensive pressure range of 6–15 GPa^20^. Furthermore, considerable D/H isotope effects were observed at the pressure at which the change was observed, which indicated that the H-bond plays an important role^19^. However, while these changes have been explained by symmetrization, the pressure conditions do not coincide with the theoretically predicted symmetrization pressure. Although attempts have been made to explain the cause of these phenomena from the structural viewpoint^21,22^, the origin remains unclear.

In this study, we conducted neutron diffraction experiments on the hydrous mineral δ-AlOOH(D), under pressures of up to 18.1 GPa and observed H-bond symmetrization for the very first time. The scattering length of hydrogen for neutrons is significant as compared to that of X-rays; thus, its contribution to the diffraction intensity is adequate to locate the hydrogen in the structure. Direct observation of the pressure response of the H-bond geometry in δ-AlOOH and its deuterated analogue provides the implications of the effects of symmetrization on the physical properties of minerals in the Earth’s mantle.

### Phase transition in δ-AlOOH and proton distribution

**Figure 2:**
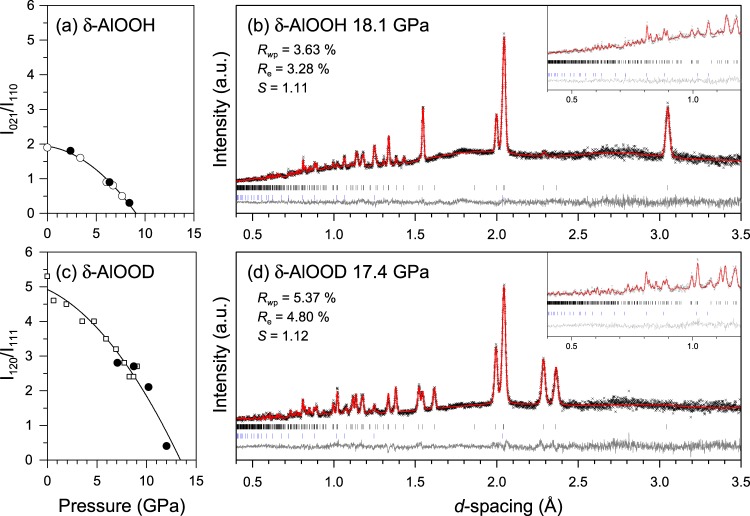
Pressure evolutions of normalized intensities of selected reflections showing rapid decrease and Rietveld fits of profiles obtained at high pressures. The symbols in (**a**) and (**c**) correspond to the results of this study (circles) and previous study^22^ (squares). The open and filled circles in (**a**) indicate the individual experimental run. The peak intensities in (**a**) and (**c**) are normalized by the maximum intensity in δ-AlOOH and δ-AlOOD, respectively. The crosses, red lines, and gray lines in (**b**) and (**d**) represent the observed, modeled, and difference profiles, respectively. The models used in the refinements are the centered H-bond model in (**b**) and the disordered H-bond model in (**d**). The vertical bars below the profiles indicate the calculated peak positions of the δ-AlOOH(D) (top) and diamond (bottom) that were used as anvils.

The neutron diffraction patterns of δ-AlOOH were collected upon compression to 18.1 GPa at room temperature by using a Paris–Edinburgh press. Because of the strong incoherent scattering of hydrogen, the background is slightly higher in δ-AlOOH compared to δ-AlOOD, but diffraction peaks are clearly seen even at small *d*-spacing below 1 Å (Supplementary Fig. S1, Fig. 2b, inset). All profiles can be fitted using the δ-AlOOH and the diamond that were used as anvils. In the first step, the atomic positions were refined using the H-ordered model with the space group of *P*2_1_*nm* (Fig. 1a), which is stable at ambient pressure, for the whole pressure range. Supplementary Fig. S2 shows the difference of interatomic distances of equatorial Al-O2 and Al-O1 of AlO_6_ octahedron. At ambient pressure, Al atom sits at the off-centered position of the AlO_6_ octahedron reflecting the fact that O1 forms H…O1 hydrogen bond while O2 forms O2-H covalent bond. The difference is over 0.1 Å at ambient pressure but it becomes negligible above 9 GPa. This result implies that the electrostatic charge becomes comparable between the O1 and O2 site and the donor and accepter oxygen atoms of the hydrogen bond are indistinguishable from each other at high pressure.

In addition, we observed a slight modification of the profiles under high pressures, suggesting a phase transition. Figure 2a shows the pressure evolution of the intensity of the 021 reflection at around 1.7 Å normalized against the 110 reflection, which has the maximum intensity. This peak has only 2% intensity compared to the maximum one under ambient conditions, but importantly, its intensity decreases continuously as the pressure increases. The critical pressure at which the intensity becomes zero was found to be 9.0 GPa by fitting the pressure evolution to a quadratic function. This additional extinction condition of *k* + *l* ≠ 2n for 0*kl* can be attributed to the transition to *Pnnm*, which is a direct super group of *P*2_1_*nm*, as pointed out in our previous single-crystal X-ray diffraction study^21^. The transition pressure is comparable roughly to the value in the previous study, where the transition occurred between 6.1 and 8.2 GPa.

**Figure 3:**
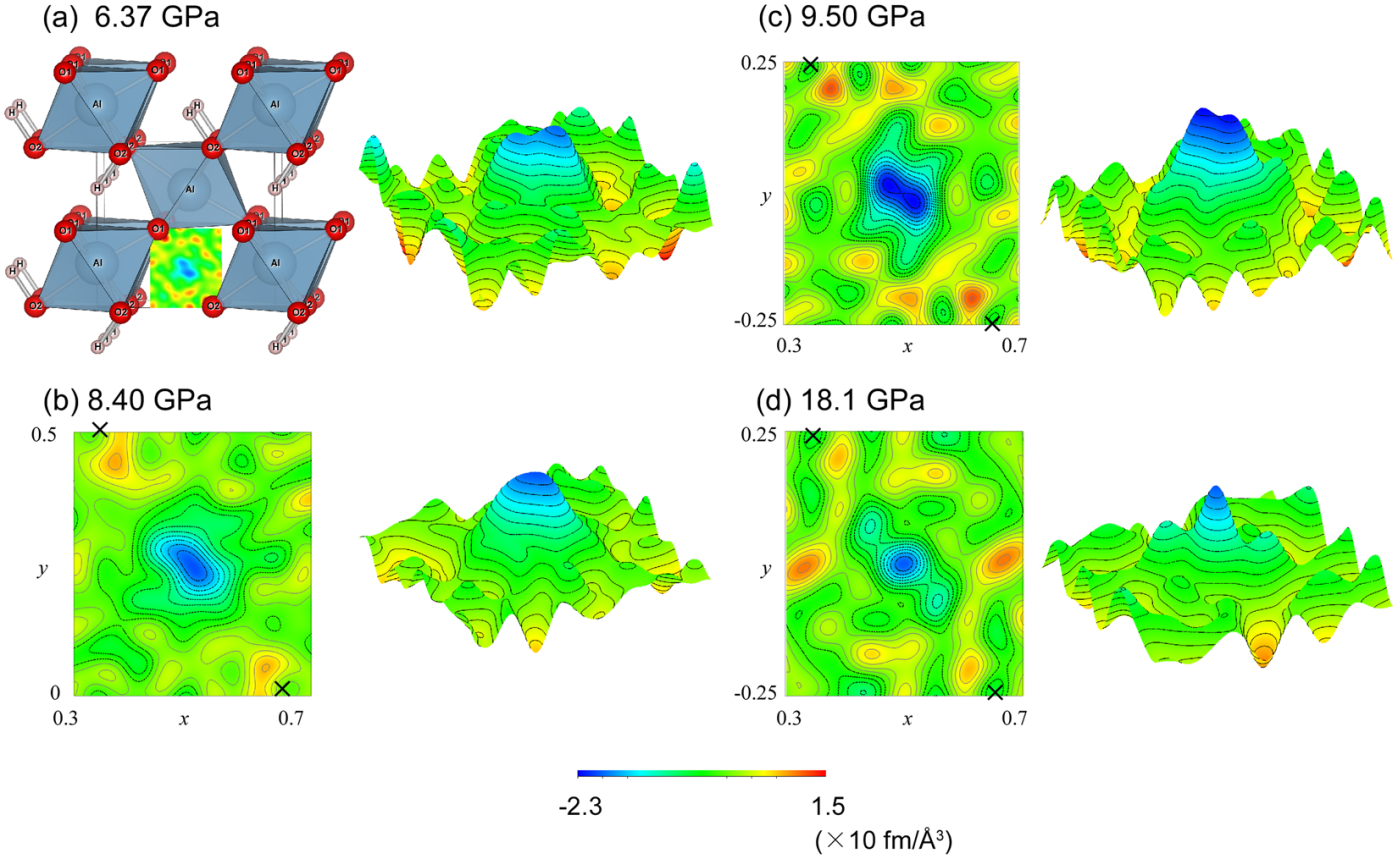
Difference Fourier maps of δ-AlOOH in section containing H-bond, showing changes in proton distribution under high pressure. The maps were generated from the difference between the observed pattern and the refined structural model without hydrogen. The hydrogen atoms in the crystal structure in (**a**) that overlap with the density map are not shown. The crosses in maps (**b**–**d**) indicate the positions of oxygen atoms. Bird’s eye views of the maps are also shown on the right-hand sides of (**a**–**d**). The contour interval is 0.2, and the zero and negative contours are shown by black dashed lines and the positive contours are shown by gray solid lines. The space groups of the model are *P*2_1_*nm* in (**a,b**) and *Pnnm* in (**c,d**).

The transition to *Pnnm* indicates that all oxygen atoms in the structure become crystallographically equivalent. By contrast, in the *P*2_1_*nm* model, there are two distinct oxygen sites corresponding to the donor and the acceptor of the H-bond. However, the space group of *Pnnm* does not require the hydrogen position to be centered. As suggested by a previous work on ice^23,24^, it is likely that the hydrogen is disordered between two equivalent sites across the center (Fig. 1b) before the symmetrization (Fig. 1c). In this configuration, both sites of oxygen atoms are in the same environment, and the structure of δ-AlOOH can be also described as *Pnnm*. To investigate the distribution of hydrogen in detail, Fourier maps were synthesized from the differences between the observed and the refined models without hydrogen atoms (Fig. 3). In these maps, hydrogen is recognized as a negative peak because it has a negative neutron scattering length.

At ambient pressure, nuclear density around the hydrogen position shows an asymmetric distribution consisting of two peaks of different heights. This feature is similar to that was observed in the case of δ-AlOOD^22,25^, and it raises the possibility that the hydrogen is partially disordered even at ambient pressure. However, refinement of the partially disordered model did not yield a reasonable result, probably because of the strong correlations between atomic positions, occupancies, and thermal parameters of the two sites located in close proximity. This result is inconsistent with the NMR study that shows a single well-defined O…O distance for proton site in δ-AlOOH^26^. To further elucidate the partially disordered model, other methods − such as optical or neutron spectroscopic studies are necessary. The position of the higher peak agrees well with the refined hydrogen position in the hydrogen-ordered model; thus, the following results are not affected by application of either the ordered or the partially disordered hydrogen model in the refinement.

A remarkable change was found in the proton distribution under high pressures. Under compression, the two peaks gradually moved toward the center between the two oxygen atoms. The difference in the heights of the two peaks decreased at the same time. At 8.40 GPa, which is just below the transition pressure, the two peaks almost merge and show a broad and asymmetric distribution (Fig. 3b). Above the transition pressure, the density peaks yield bimodal distribution with the same peak height (Fig. 3c), clearly indicating that the *P*2_1_*nm* to *Pnnm* transition can be interpreted as complete disordering of the H-bond rather than symmetrization. Further compression to 18.1 GPa led to merging of the distribution into a single peak at the center of the two oxygen atoms, suggesting that hydrogen symmetrization takes place at least the pressure between 18.1 GPa and 16.1 GPa (Fig. 3d). These observations revealed that there are two transitions in δ-AlOOH, namely, disorder of hydrogen bond at 9.0 GPa accompanied by a change in the space group, which is probably the onset of tunneling as discussed later, and symmetrization at 18.1 GPa without any change in the space group.

### D/H Isotope effect on phase transition

**Figure 4:**
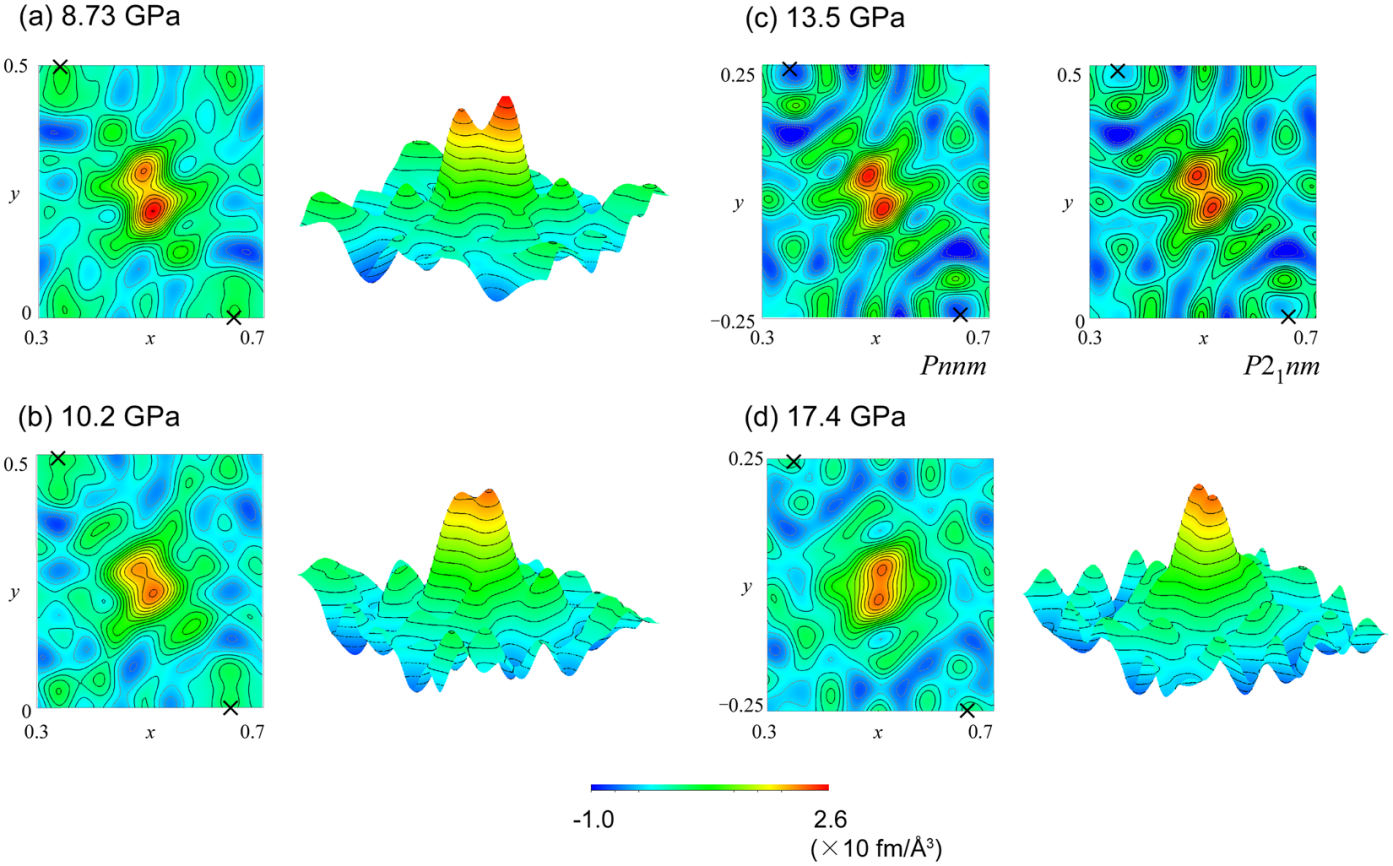
Difference Fourier maps of δ-AlOOD in section containing H-bond. Difference Fourier maps generated from the difference between the observed pattern and the refined structural model without deuterium. The contour interval is 0.2, and the zero and negative contours are denoted by black dashed lines and positive contours are denoted by gray solid lines. The space groups of the model are *P*2_1_*nm* in (**a,b**), *Pnnm* in (**d**) and the comparison of the maps generated using two models are shown in (**c**).

To investigate the isotope effect on the transition, neutron diffraction patterns of δ-AlOOD were measured at 17.4 GPa (Fig. 2d). Different scattering lengths of deuterium from hydrogen make the intensity profiles completely different; for example, the 021 reflection becomes invisible in the deuterated sample. We used the 120 reflection instead, which leads to zero intensity in *Pnnm*, as a criterion of the transition. The intensity becomes smaller under high pressures (Fig. 2c, Supplementary Fig. S1d), and the transition pressure was found to be 13.4 GPa in the case of δ-AlOOD, which is 4.4 GPa higher than that of δ-AlOOH. Figure 4 shows the difference Fourier maps of δ-AlOOD at selected pressures. In this case, deuterium appears as a positive nuclear density peak in the map because it has a positive scattering length. In accordance with the previous neutron diffraction study^22^, the density peak in the Fourier map shows asymmetric bimodal distribution under lower pressures. With increasing pressure, the peaks move toward the center between the two oxygen atoms (Fig. 4a–b). Above the transition pressure, the distribution has a symmetric bimodal shape (Fig. 4c), indicating that the transition is defined by the disorder of deuterium as well as that of δ-AlOOH. Under pressures of up to 17.4 GPa, the distribution remained bimodal and did not merge into a single peak in the case of δ-AlOOD (Fig. 4d).

### Evolution of H-bond geometry under high pressures

**Figure 5:**
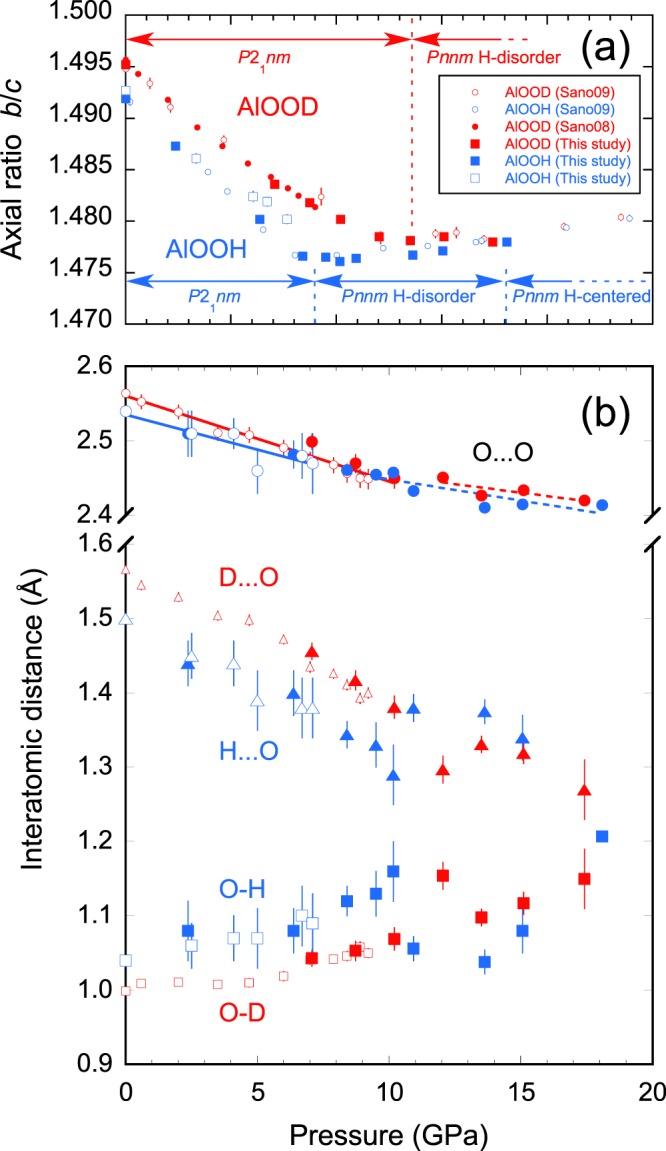
(**a**) Pressure evolution of axial ratio *b*/*c* and (**b**) H-bond geometries of δ-AlOOH (blue symbols) and δ-AlOOD (red symbols). The small symbols indicate the results of previous studies on δ-AlOOH(D)^19,22^.

Based on the models determined as described above, the structures, including hydrogen positions under high pressures, were refined. Figure 5(b) shows the pressure evolutions of the O-H(D)···O geometry. The O···O distances decrease almost linearly towards the pressures of the disorder transitions, but the rates of this change appear to decrease slightly above the transition pressures. The decrease in the O···O distances results in strengthening of the H(D)···O H-bond and weakening of the O-H(D) covalent bond, as reflected by the decrease and increase in each of these distances. This result agrees with the result of a previous high pressure IR study, in which softening of the OH stretching bond was demonstrated^27^. The pressure evolutions of the O-H(D) and H(D)···O distances are non-linear, and the rate of change is accelerated toward disorder and symmetrization. Specifically, changes in the O-H bond length are remarkable, and it was 0.12 Å under compression of 10.2 GPa that corresponds to 11% increase. This rate is one to two orders of magnitude higher than that of other known materials. In general, compression of the O···O distance results in decreased length of the H-bond, while its effect on the length of the covalent bond is known to be small. For instance, the evolution of the O-D bond length is reported to be linear with small gradients of 0.0004 Å/GPa for ice VIII under 10 GPa^28^ and 0.004 Å/GPa for ice VII under 5.4 GPa^29^. The large changes in the covalent bond length that were observed in this study can be considered as a characteristic feature of the pressure response of the strong H-bond.

A comparison of the H-bond geometries of δ-AlOOH and δ-AlOOD under the same pressure revealed the presence of a significant isotope effect. Deuteration leads to an increase in the O···O and the hydrogen bond lengths, and a decrease in the covalent bond length under pressures lower than 10 GPa. The difference in bond length observed in this study is in the range of the known geometric isotope effect^30^. An investigation of the H-bond geometries of various materials under ambient pressure suggests that the O···O distance increases by about 0.02~0.03 Å upon deuteration, where the O···O distance is around 2.5 Å. Above 10 GPa, there was an anomalous increase in the H(D)-bond length and shortening of the covalent bond in both δ-AlOOH and δ-AlOOD. At the moment, we cannot interpret this phenomenon properly, and whether this is true for H-bonds in general or specific to δ-AlOOH needs to be clarified in future studies. Further compression results in shrinkage of the H-bond geometry and, eventually, symmetrization at 18.1 GPa in the case of δ-AlOOH. The O···O distances were determined to be 2.455(5) Å at the disorder and 2.414(6) Å at the symmetrization in δ-AlOOH. The O···O distance at which deuterium is disordered is 2.427(3) Å, which is slightly shorter than that for hydrogen. It has been shown that deuteration shifts the disorder transition, and probably the symmetrization as well, to a higher pressure.

The symmetrization pressure of δ-AlOOH was about 12 GPa lower than that predicted in a previous theoretical study^10^. Probably, the reasons for this discrepancy are quantum and temperature effects that were not included in the calculations, as mentioned in that study. Theoretical studies on ice have pointed out that the symmetrization pressure decreases significantly when the quantum effect is considered^24^. Compared to other materials such as ice and hydrates, the symmetrization pressure of δ-AlOOH is considerably low. The short O···O distance under ambient pressure and effective compression of the O···O distance between the AlO_6_ octahedral chains is considered to contribute to lowering of the transition pressure.

In the case of ice, a few different states towards the symmetrization have been proposed by theoretical calculations, namely, statistically disordered ice VII; dynamically disordered ice VII, in which the proton tunnels through the double minimum potential well and takes bimodal distribution; and ice X in which proton is centered between two oxygen atoms. Two types of ice X are also proposed; ice X’ in which the proton takes a broad and unimodal distribution at the center when its lowest vibrational level overcomes the potential barrier of the double well, and ice X in which the proton potential finally become single minimum^24^. The term symmetrization here corresponds to the transition from dynamically disordered ice VII to ice X’ that proton is centered. The O···O separation in δ-AlOOH at 18.1 GPa (2.414 Å) shows good agreement with that of ice at the transition pressure to the ice X’ of around 60 GPa (2.4 Å)^6,7^. Thus, the symmetrization observed in the present study is very likely to correspond with that of ice X’. Likewise, the disorder transition observed at 9.0 GPa in δ-AlOOH and 13.4 GPa in δ-AlOOD could be interpreted as the dynamically disordered state by the proton tunneling. This is also supported by the observed isotope effect on the transition pressures because the mass effect is significant in the tunneling and zero-point vibration thus deuteration induces the shift of the transition pressures to higher. It should be noted that the diffraction method determines the average structure; thus, from the results of the present study, it is difficult to distinguish whether the hydrogen is statistically disordered or dynamically disordered. Further investigation, for example, neutron diffraction and spectroscopic study at low temperature where the zero-point vibration is dominant, will provide better understanding of the different disordered states in future.

### Stiffening induced by the H-bond disorder and symmetrization under mantle conditions

Few hydrous minerals are stable under the Earth’s lower mantle conditions, such as phase D^31^, δ-AlOOH^15^, its isomorph of magnesium silicate phase H^17^, and FeOOH^32,33^. Theoretical calculations have indicated that all the H-bonds in these minerals become symmetric under the conditions of the lower mantle^10–12,16–18^. Hence, it is of paramount importance to consider how the symmetrization process affects the physical properties of minerals. The present neutron diffraction study has shown that the hydrogen is not centered but dynamically disordered between pressures of 9.0 GPa to at least 15.1 GPa in the case of δ-AlOOH. Above the disorder pressure, the O···O separation of the H-bond becomes slightly less compressible (Fig. 5b). Accordingly, variation of the rotation angle of the AlO_6_ octahedra, which is the main compression mechanism in *a*-*b* plane below the transition pressure, also decreases (Supplementary Fig. S4). This modification in the compression mechanism explains the change in axial compressibility, axial ratio (Fig. 5a), increase in bulk modulus^19^, and anomalous increase in sound velocities^20^, which were reported previously and had been interpreted as symmetrization of the H-bond. The present neutron diffraction study has pointed out the importance of the H-bond disorder as a precursor of the symmetrization in the physical properties of minerals under high pressures.

Finally, it is noteworthy that as the present study was conducted under ambient temperature, we should consider the temperature effect when applying the results to the conditions in the Earth’s mantle. Temperature can affect the symmetrization in two contradictory ways: the expansion of the lattice leads to an increase in the O···O distance as well as an increase in thermal vibration. The first effect increases the disorder and symmetrization pressure, whereas the latter causes it to decrease. The transition pressures are determined by the dominant effect. For example, in case of phase D, the pressure at which the axial ratio changes is shifted upward by 3 GPa as the temperature increases to 1300 K^34^; thus, thermal expansion is considered dominant in this case. Assuming the thermal expansion of δ-AlOOH is of the same order as that in phase D, the disorder, and probably, the symmetrization pressure would increase by several GPa under the conditions in the Earth’s mantle. Previous studies reported that δ-AlOOH can be formed by the decomposition of phase Egg (AlSiO_3_OH), which is possible water carrier in subducting sediment layers of slabs^35^ and is found in inclusions of diamond^2^, at around 22 GPa. Consequently, the H-bond in δ-AlOOH is considered to be symmetric in the entire stability field in the mantle conditions.

## Methods

The samples of δ-AlOOH and δ-AlOOD were synthesized at 18 GPa and 900 °C by using a Kawai-type multi-anvil press. The samples used herein were identical to those used in previous studies, and details of the synthesis are presented elsewhere^21,22,25^. The samples were sealed into TiZr encapsulating gaskets^36^ with deuterated 4:1 methanol–ethanol pressure-transmitting medium and loaded into a Paris–Edinburgh cell^37^. Time-of-flight neutron powder diffraction experiments were conducted at the SNAP diffractometer in the SNS at Oak Ridge National Laboratory and at the PLANET diffractometer^38^ in the Material and Life Science Facility at J-PARC. In the experiment at SNAP, cubic boron nitride anvils were used. The cell was placed such that the compression axis was perpendicular to the beam. The incident beam was introduced into the cell through the gasket, and the scattered neutrons were detected through the gasket by means of a detector fixed at 2θ = 89.5°. The accelerator power of the SNS was 1 MW. In the experiment at PLANET, sintered diamond anvils were used. The cell was placed such that the compression axis was aligned coaxially with the beam, and the incident beam was introduced to the sample through the anvil. The scattered neutrons were detected through the gasket with 90° detector banks. The accelerator power of the J-PARC was 300 kW. Radial collimators with gauge volumes of 3 mm and 1.5 mm were used in the experiments on δ-AlOOD and δ-AlOOH, respectively, to reduce background.

Diffraction patterns were collected for 4–7.5 h at several oil pressures upon compression. Pressures were calculated using the unit-cell volume of δ-AlOOH and δ-AlOOD based on third-order Birch–Murnaghan equation of states^19^. Slight broadening of the peaks was observed under high pressure, but it was not significant in the refinement; for example, the peak widths under ambient pressure and 18.1 GPa were 0.77% and 0.91% in Δ*d*/*d*, respectively, for the data of δ-AlOOD obtained at the PLANET. The axial ratio of b/c of the present study is well consistent with that of the previous study conducted under quasi-hydrostatic condition using He and Ne as a pressure medium (Fig. 5b), suggesting that the effect of deviatoric stress is small. Test refinement of δ-AlOOH using deuterium and hydrogen at the hydrogen site resulted in no occupation of deuterium, showing that D-H isotope exchange did not occur between the sample and the deuterated pressure medium during the experiment.

Profiles of vanadium in the high-pressure cell were also collected at the same load with the sample measurement. The empty cell profile was subtracted from both the sample and the vanadium data then the intensity of the sample profile was normalized with that of vanadium to correct the energy profile of the incident neutron beam, the attenuation of the cell and the radial collimators, and the detector efficiency. The structure was refined by means of Rietveld method^39^ using GSAS^40^ and EXPGUI software^41^. The peak profile parameters were first refined using the Lebail method and then fixed during subsequent Rietveld refinement cycles. The scale factor, background functions, lattice parameters, atomic positions, and isotopic displacement parameters were refined. The isotopic displacement parameters of the two oxygen atoms in the *P*2_1_*nm* model were constrained to have the same value. In the experiments using sintered diamond anvils, the diffraction peaks of diamond were observed; thus, the lattice parameter of diamond was also refined. In the refinement of δ-AlOOD, the occupancy of deuterium and hydrogen were fixed to 0.744 and 0.266, as determined previously under ambient conditions^25^. The data obtained at SNAP, in which the *d*-spacing ranges from 0.46 Å to 2.31 Å with constant binning of 30 μs, and that obtained at PLANET, in which the *d*-spacing ranges from 0.32 Å to 3.70 Å with constant binning of 10 μs, were used in the Rietveld refinement. The difference Fourier maps in Fig. 3 and 4 were drawn using VESTA^42^. For the synthesis of the difference Fourier maps, the data obtained at PLANET was used which could access to larger *q*_max_ to get good resolution. The comparison of the resolution of Fourier map using different *q*_max_ is shown in the Supplementary Fig. S5.

## Electronic supplementary material

Dataset 1
