# Supplementary material for: Direct observation of symmetrization of hydrogen bond in δ-AlOOH under mantle conditions using neutron diffraction
Source: Sci Rep. 2018 Oct 19;8:15520. doi: 10.1038/s41598-018-33598-2 (PMC6195538; doi:10.1038/s41598-018-33598-2)
Supplement: Supplementary file 1 — Dataset 1 [file 41598_2018_33598_MOESM1_ESM.docx]

Direct observation of symmetrization of hydrogen bond in δ-AlOOH under mantle conditions using neutron diffraction

Supplementary information

Asami Sano-Furukawa^1*^, Takanori Hattori^1^, Kazuki Komatsu^2^, Hiroyuki Kagi^2^, Takaya Nagai^3^, Jamie J. Molaison^4^, António M. dos Santos^4^ and Christopher A. Tulk^4^

^1^ J-PARC Center, Japan Atomic Energy Agency, Tokai-mura, Ibaraki 319-1195, Japan.

^2^ Geochemical Research Center, Graduate School of Science, The University of Tokyo, Tokyo 113-0033, Japan.

^3^ Department of Natural History of Sciences, Faculty of Science, Hokkaido University, Sapporo 060-0810, Japan.

^4^ Neutron Scattering Division, Oak Ridge National Laboratory, Oak Ridge, Tennessee 37831, U.S.A.

* Correspondence to sanoasa@post.j-parc.jp

**Supplementary Figure S1**


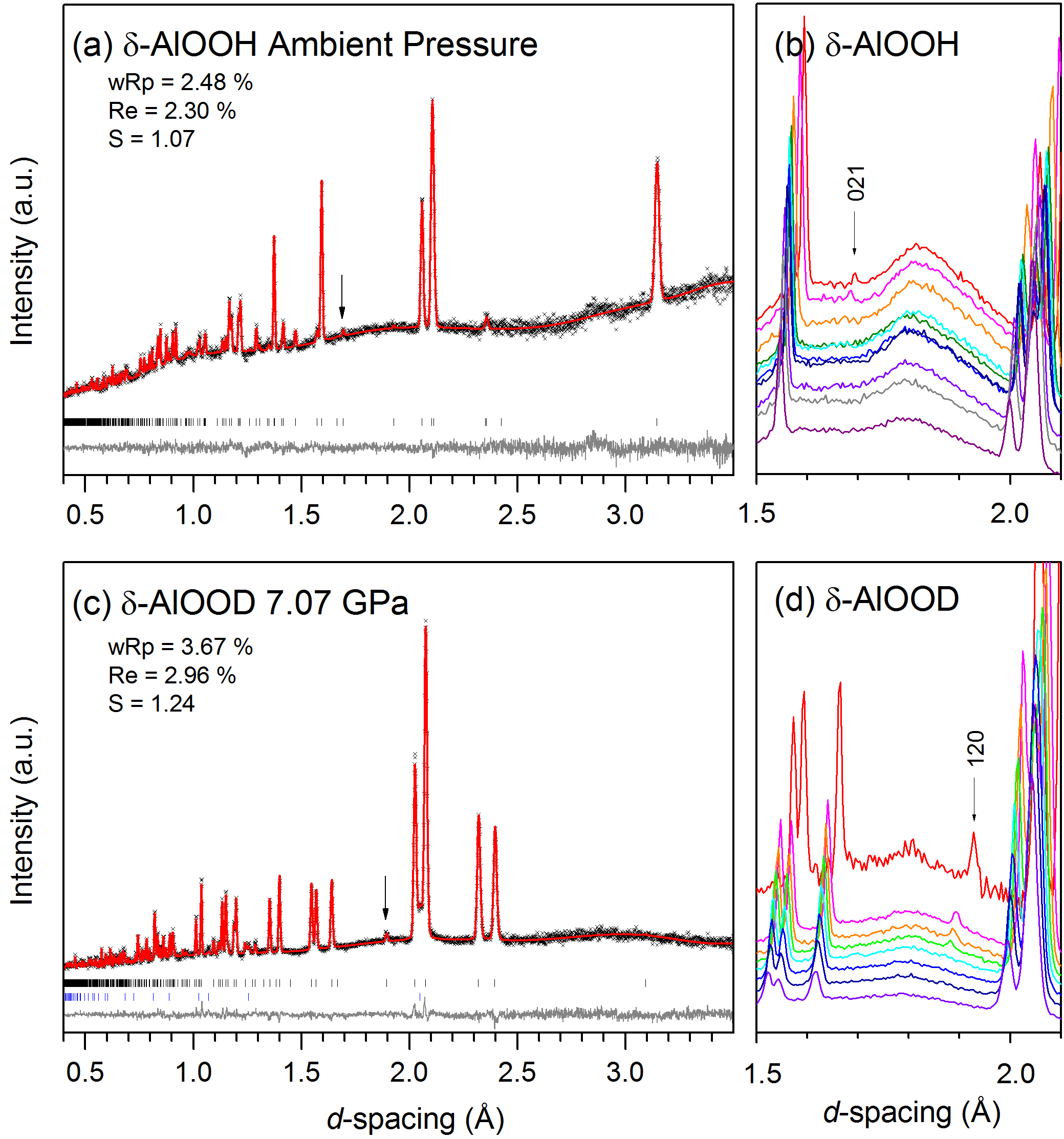


**Supplementary Figure S1: Rietveld fits of profiles of (a) δ-AlOOH and (c) δ-AlOOD with *P*2_1_*nm* model and (b, d) enlarged raw profile around *d*-spacing of 2 Å.** The allows in (a) and (c) indicate the reflections of 021 and 120, respectively. The measurement conditions were ambient pressure, 2.37, 6.37, 8.40, 9.50, 10.2, 10.9, 13.6, 15.1 and 19.1 GPa for (b) and ambient pressure, 7.07, 8.73, 10.2, 12.0, 13.5, 15.1 and 17.4 GPa for (d) from top to bottom, respectively. The binning of data of (b, d) was Δt = 40 μs to get a good statistics.

**Supplementary Figure S2**

**
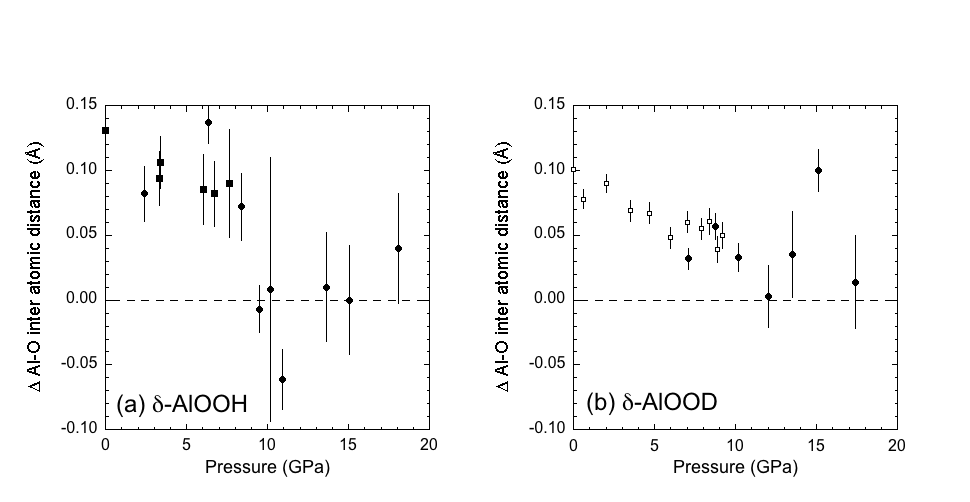
**

**Supplementary Figure S2: Difference of interatomic distances between Al-O2 and Al-O1 at equatorial plane of AlO_6_ octahedron.** All the data was refined with *P*2_1_*nm* model. The open symbols in (b) represent the results of the previous study^1^. The filled symbols are the results of the present study indicating the individual experimental runs with different symbols.

**Supplementary Figure S3**

**
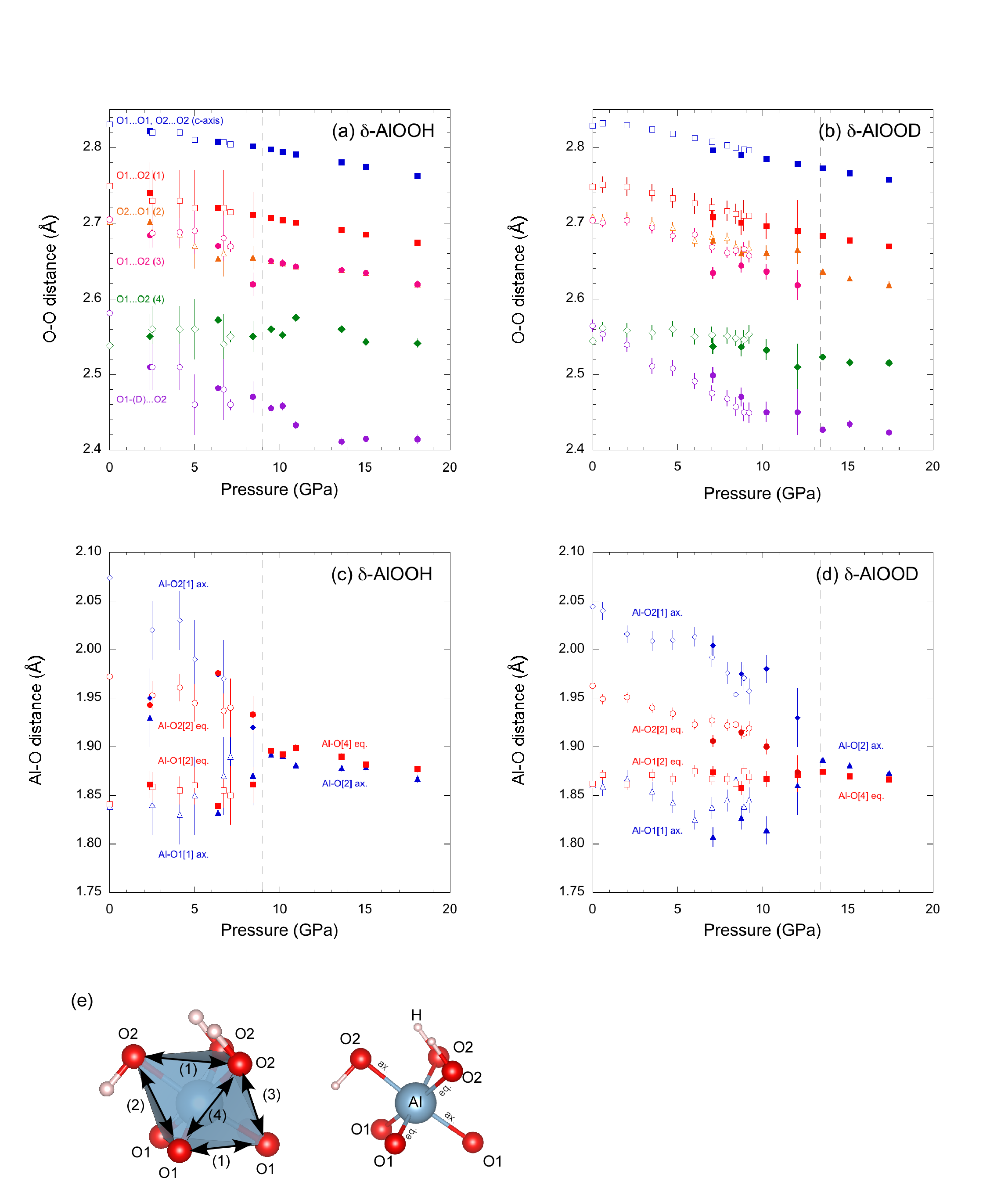
**

**Supplementary Figure S3: (a, b) Variations of O-O distances, and (c, d) axial and equatorial Al-O bond lengths of AlO_6_ octahedron in δ-AlOOH and δ-AlOOD.** The numbers in the parentheses in the captions of (a) and (b) corresponding to these variations are indicated by the octahedral edge in (e). The numbers in the parentheses in the captions of (c) and (d) indicate the numbers of identical bonds in the octahedra. The open symbols in (b) and (d) represent the results of a previous study^1^, and the other symbols are those used in the present study. Results of Rietveld fitting with space group of *P*2_1_*nm* and *Pnnm* at below and above the transition pressure are shown. The gray dotted line indicates the transition pressure from *P*2_1_*nm* to *Pnnm*.

**Supplementary Figure S4**

**
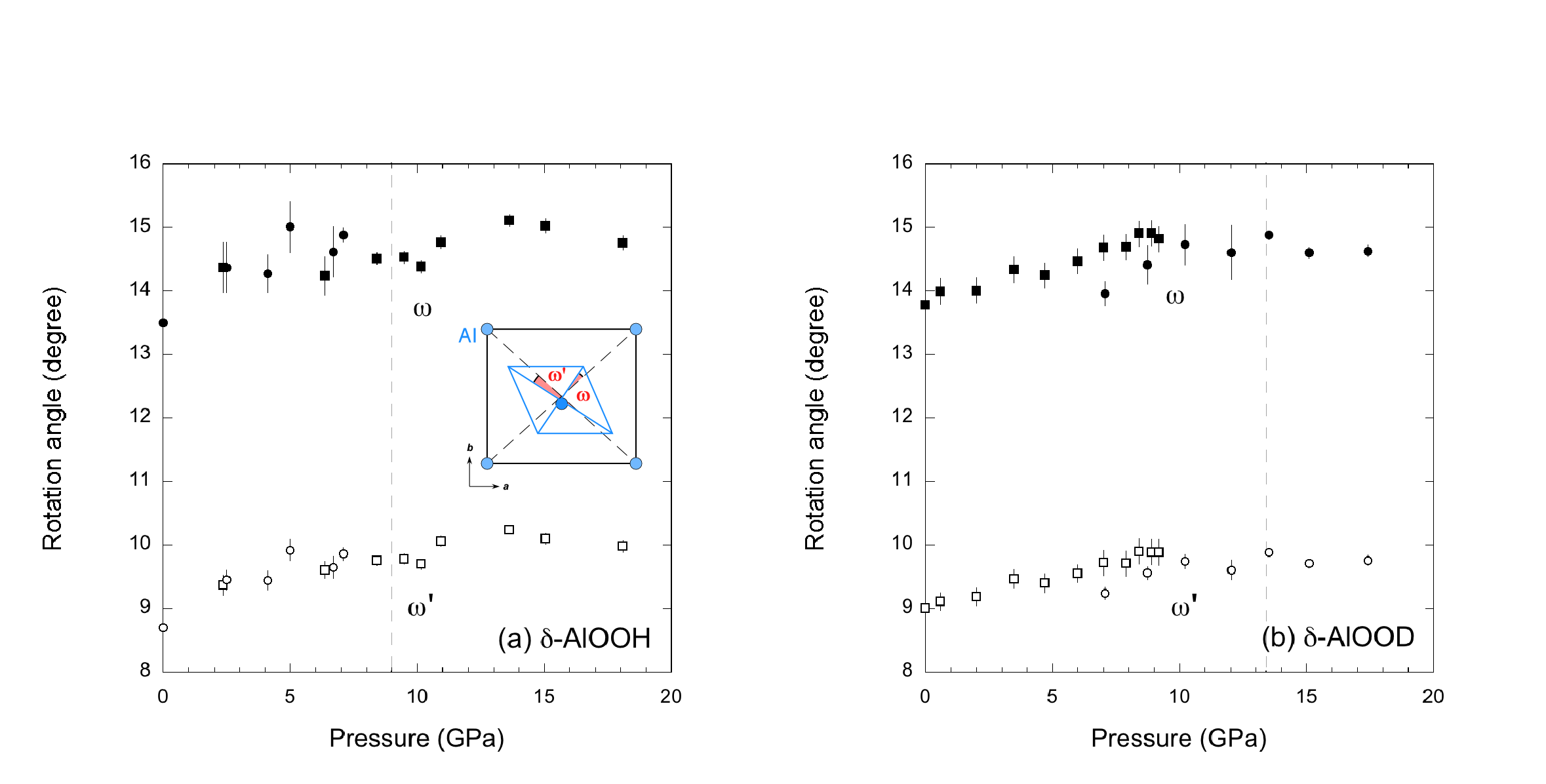
**

**Supplementary Figure S4: Variations of rotation angles ω and ω’ of AlO_6_ octahedra in (a) δ-AlOOH and (b) δ-AlOOD.** The gray dotted line indicates the transition pressure from *P*2_1_*nm* to *Pnnm*.

**Supplementary Figure S5**

**
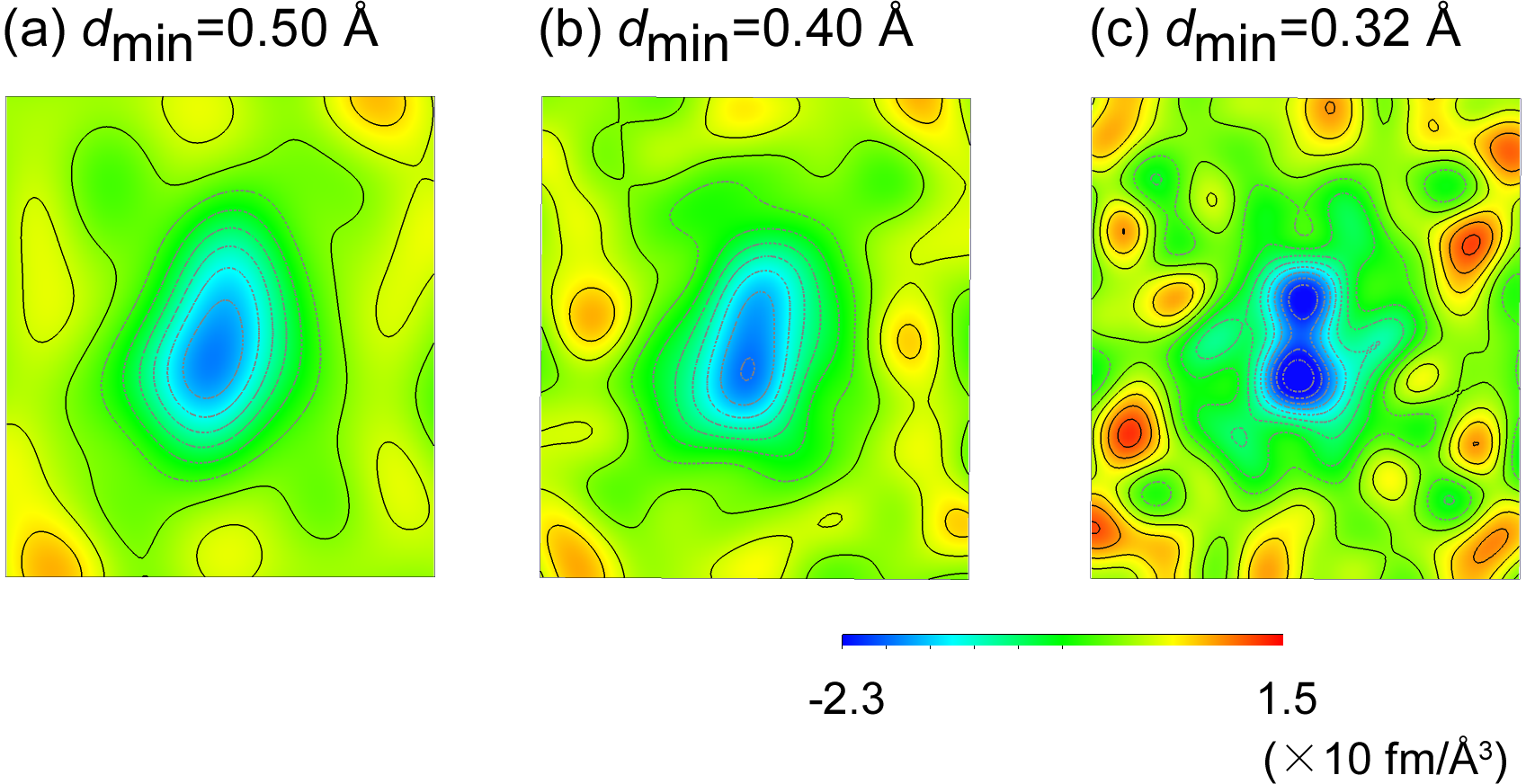
**

**Supplementary Figure S5: Difference Fourier maps of δ-AlOOH in section containing H-bond at ambient pressure using different *d*-spacing range.** The resolution of the Fourier map is determined by the *Q*_max_, which was used to synthesize. The maps become blurred when the *Q*_max_ become small, which corresponds to the larger *d*-spacing (a, b). In the present study, the *d*-spacing ranges to 0.32 Å (c) was used in the discussion.

**Supplementary Table S1: Details of structure refinements at selected pressures.**

| Sample | δ-AlOOH |  |  | δ-AlOO(D_0.744_H_0.256_) | |
| --- | --- | --- | --- | --- | --- |
| Pressure (GPa) | 6.37 | 9.50 | 18.1 | 10.2 | 17.4 |
| Space group | *P*2_1_*nm* | *Pnnm* | *Pnnm* | *P*2_1_*nm* | *Pnnm* |
| *a* (Å) | 4.6888(2) | 4.6326(2) | 4.5774(3) | 4.6148(2) | 4.5700(4) |
| *b* (Å) | 4.1964(2) | 4.1303(2) | 4.0829(4) | 4.1222(2) | 4.0763(6) |
| *c* (Å) | 2.8215(2) | 2.7973(2) | 2.7625(3) | 2.78487(8) | 2.7579(2) |
| *V* (Å^3^) | 54.338(3) | 56.524(3) | 51.628(5) | 52.977(2) | 51.377(6) |
| Refinement |  |  |  |  |  |
| *R*_p_ | 2.67 | 2.56 | 3.31 | 3.37 | 4.92 |
| *R*_wp_ | 2.99 | 2.87 | 3.59 | 3.46 | 5.38 |
| *R*_e_ | 2.17 | 2.53 | 3.30 | 3.14 | 4.08 |
| *S* | 1.12 | 1.14 | 1.09 | 1.21 | 1.12 |
| Data points | 3193 | 3193 | 3193 | 3194 | 3194 |
| Parameters | 31 | 27 | 25 | 29 | 23 |

Notes: *R*_WP_ = Σ*_i_*|*y_i_-f_i_(x*)|/ Σ*_i_y_i_*, *R*_P_ = [Σ*_i_w_i_*{*y_i_-f_i_(x*)}^2^/ Σ*_i_w_i_y_i_^2^*]^1/2^, *R*_e_ = [*N-P*/ Σ*_i_w_i_y_i_^2^*]^1/2^, *S* = *R*_WP_/*R*_e_ , where *y_i_*, observed intensities; *f_i_(x)*, calculated intensities; *w_i_*, statistical weight; *N*, Number of data points; *P*, Number of parameters.

**Supplementary Table S2 Atomic positions, isotropic displacement parameters, and H-bond geometry at selected pressures.**

|  | δ-AlOOH |  |  | δ-AlOO(D_0.744_H_0.256_) | |
| --- | --- | --- | --- | --- | --- |
| Pressure (GPa) | 6.37 | 9.50 | 18.1 | 10.2 | 17.4 |
| Spacegroup | *P*2_1_*nm* | *Pnnm* | *Pnnm* | *P*2_1_*nm* | *Pnnm* |
|  |  |  |  |  |  |
| Al |  |  |  |  |  |
| *x* | 0 | 0 | 0 | 0 | 0 |
| *y* | 0.280(3) | 0 | 0 | 0.267(2) | 0 |
| *z* | 0 | 0 | 0 | 0 | 0 |
| *U*_iso_×100 (Å^2^) | 0.54(16) | 1.22(14) | 0.88(17) | 0.66(7) | 0.46(9) |
| O1 |  |  |  |  |  |
| x | 0.344(3) | 0.3465(5) | 0.3464(6) | 0.338(3) | 0.3478(5) |
| y | 0.495(2) | 0.2423(8) | 0.2402(10) | 0.492(1) | 0.2429(7) |
| z | 0 | 0 | 0 | 0 | 0 |
| *U*_iso_×100 (Å^2^) | 0.41(4) | 0.42(3) | 0.59(5) | 0.33(2) | 0.44(4) |
| O2 |  |  |  |  |  |
| x | 0.652(4) |  |  | 0.641(3) |  |
| y | 0.008(2) |  |  | 0.0040(12) |  |
| z | 0 |  |  | 0 |  |
| *U*_iso_×100 (Å^2^) | 0.41(4) |  |  | 0.33(2) |  |
| H/D |  |  |  |  |  |
| g | 1 | 0.5 | 1 | 1 | 0.5 |
| x | 0.523(5) | 0.479(4) | 1/2 | 0.508(3) | 0.493(7) |
| y | 0.225(4) | 0.013(5) | 0 | 0.217(2) | 0.012(8) |
| z | 0 | 0 | 0 | 0 | 0 |
| *U*_iso_×100 (Å^2^) | 2.2(2) | 1.29(19) | 1.8(2) | 1.44(11) | 2.1(2) |

Notes: The isotropic atomic displacement parameters in the *P*2_1_*nm* model of oxygen were constrained to be *U*_iso_(O1) = *U*_iso_(O2).

**Reference**

1. Sano-Furukawa, A., Komatsu, K., Vanpeteghem, C. B. & Ohtani, E. Neutron diffraction study of δ-AlOOD at high pressure and its implication for symmetrization of the hydrogen bond. *Am. Mineral.* **93**, 1558-1567 (2008).
